# Analytical confirmation of imidacloprid poisoning in granivorous Cape spurfowl (*Pternistis capensis*)

**DOI:** 10.4102/jsava.v89i0.1637

**Published:** 2018-07-09

**Authors:** Christo J. Botha, Elizabeth C. du Plessis, Heleen Coetser, Magda Rosemann

**Affiliations:** 1 Department of Paraclinical Sciences, University of Pretoria, South Africa; 2 VETPATH, Division of IDEXX Laboratories, South Africa; 3 Toxicology and Ethnoveterinary Medicine, Agricultural Research Council-Onderstepoort Veterinary Institute, South Africa; 4 National Horseracing Authority of Southern Africa, Kenilworth, South Africa

## Abstract

Reports were received that Cape spurfowl (*Pternistis capensis*) fell during flight and scrambled uncoordinatedly for cover and some died. Three carcases were submitted for necropsy examination, which revealed mainly congestion of the carcases and haemorrhages. Common causes of acute mortalities in birds were first excluded, but there was a history of possible exposure to imidacloprid-treated barley seeds. Imidacloprid, a neonicotinoid insecticide, is used to protect various crops against invertebrate pests. The combined crop contents and pooled liver samples collected from the Cape spurfowl during necropsy were submitted for liquid chromatography–high-resolution mass spectrometry (LC-HRMS) for imidacloprid analysis. Imidacloprid and several of its metabolites were detected in the samples. Farmers should cover sown seeds with a layer of soil and remove any spilled seeds, as granivorous birds are susceptible to imidacloprid intoxication. Regulatory authorities should re-evaluate the risk posed by imidacloprid-treated seeds for pollinators and granivorous birds.

## Introduction

Imidacloprid, a neonicotinoid insecticide, is used in veterinary medicine as a topical ectoparasiticide (mainly for flea control) and also as an agricultural pesticide for the control of invertebrate pests affecting crop production and ornamental flowers (Tomizawa & Casida 2005; Van Zyl 2013). Various formulations are retailed, and it is commonly used as a seed treatment (Gibbons, Morrissey & Mineau 2015). Imidacloprid acts as an agonist at nicotinic cholinergic receptors in the peripheral and central nervous system (Tomizawa & Casida 2005). The oral LD_50_ of imidacloprid in rats is 450 mg/kg, but birds are more sensitive (Tomizawa & Casida 2005). The acute oral LD_50_ for grey partridge (*Perdix perdix*) is 13.9 mg/kg and 31 mg/kg for Japanese quail (*Coturnix japonica*) (Gibbons et al. 2015; Tomizawa & Casida 2005). Balani, Agrawal and Thaker (2011) referred to an ‘apparent’ LD_50_ of 50 mg/kg in chickens.

Initially, it was reported that imidacloprid has a repellent effect that will deter granivorous birds from ingesting treated seeds (Avery, Decker & Fisher 1994). However, recently there have been several reports of wild birds being adversely affected. Millot et al. (2017) provided evidence of mortality events attributed to the ingestion of imidacloprid-treated seeds by wild birds, mainly pigeons (*Columba* species) and grey partridges, in France.

## Case history

During May 2017, reports were received of Cape spurfowl (*Pternistis capensis*) ‘acting strangely’ on open crop fields near the Overberg Renosterveld Conservancy, Greyton, Western Cape Province, South Africa. The birds would attempt to fly and then somersaulted or tumbled out of the air, dropping to the ground, crash landing and bouncing out of control. After a few seconds, they would attempt to hide, but were clearly struggling to move. Instead of flying, the birds scrambled away and hurriedly dashed for cover. The farmers from the area indicated that not only Cape spurfowl but Greywing francolin (*Francolinus africanus*) was affected too. The fields were recently sown with wheat or barley and seeds were visible on the ground. One of the farmers indicated that imidacloprid (Ronsek 600 FS, Villa Crop Protection [Pty], Ltd.) was used as a systemic insecticide seed treatment before sowing. An intoxication was suspected and three carcases of Cape spurfowl were later submitted for necropsy examination.

### Preliminary investigations

**FIGURE 1:**
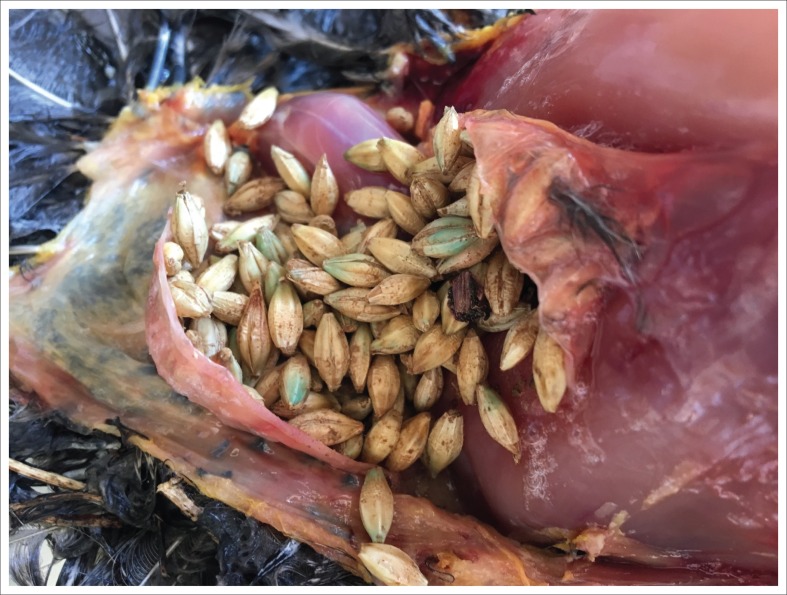
Crop contents with bluish discoloured barley seeds visible.

Macroscopically the following were noted: moderate haemorrhages present in the coelomic cavity and in the air sacs; moderate, diffuse congestion of the carcases; moderate congestion of the livers; severely enlarged and congested spleens; severe to mild pulmonary congestion and haemorrhage; and the kidneys were mildly to moderately congested. The crops of the birds were filled with blueish-stained barley seeds (Figure 1). The major gross lesions observed were indicative of acute mortality.

Primary causes of neurological signs and acute mortality in birds were ruled out first. Pooled organ samples from the birds were submitted to exclude avian influenza and Newcastle disease viruses using polymerase chain reaction (PCR) techniques. Neither Newcastle disease nor avian influenza viral infections could be detected in these birds.

Crop contents were collected for routine toxicological analysis. The combined crop contents of the birds were extracted following an approved standard operating procedure. Briefly, 20 g of combined crop contents were extracted with 100 mL of ethyl acetate on a mechanical shaker (Labcon) for 1 hour and analysed using gas chromatography–mass spectrometry (GC-MS, Varian 3900, Saturn 2100T). No common pesticide for which routine analysis was performed (i.e. organophosphorus, carbamate and organochlorine compounds or phosphine gas) was identified in the crop contents.

### Analysis of samples for imidacloprid

As there was a history of possible exposure to imidacloprid, the extracted and evaporated crop content sample (as prepared and used for pesticide analysis) and a pooled liver sample were submitted for further chemical analysis.

The samples were analysed with liquid chromatography–high-resolution mass spectrometry (LC-HRMS) to determine the presence of imidacloprid.

A standard containing 1 mg/mL imidacloprid (Sigma-Aldrich Chemie, Germany) was dissolved in acetonitrile (Burdick & Jackson, USA) and used to prepare a calibration curve in methanol at 0 ng/mL, 50 ng/mL, 100 ng/mL, 250 ng/mL, 500 ng/mL, 1000 ng/mL, 2000 ng/mL, 5000 ng/mL and 10 000 ng/mL. The calibrators were injected into the LC-HRMS. Chicken liver was used to prepare blank liver and spiked liver (50 ng/g) quality control (QC) samples.

The extracted crop sample container was rinsed with acetonitrile and centrifuged, and the clear layer was decanted and then evaporated. The sample was reconstituted in 200 *μ*L methanol and injected into the LC-HRMS instrument. The sample was further diluted to fall within the calibration range.

The pooled liver sample was chopped, and duplicate 5 g portions were weighed for analysis. The liver samples were homogenised (IKA Ultraturrex homogeniser, Zymark Turbovap) with 10 mL acetonitrile and centrifuged at 3000 g for 15 min, and the clear layers were evaporated and reconstituted in 1 mL methanol. The extracts were injected into the LC-HRMS instrument.

The same LC-HRMS conditions described in previous toxicology investigations by Botha et al. (2014) and Singo et al. (2017) were used. Full mass spectrometry (MS) experiments were used to record the initial qualitative and quantitative results. Qualitative data were processed with ToxID, and Xcalibur Quanbrowser software (Thermo Fisher, USA) was used to quantify the results. Full mass spectrometry to data-dependent mass spectrometry (FullMS > ddMS2) experiments were used to obtain spectra of the metabolite peaks and were matched, where possible, with library entries for confirmation of the identities.

### Results of the analysis

Imidacloprid was detected in all the samples analysed by LC-HRMS. In this study, 14 314 ng imidacloprid was determined in 20 g of pooled and extracted crop contents of three Cape spurfowl, equating to a concentration of 7.16 *μ*g/g. The imidacloprid concentration detected in the crop contents was comparable to previous studies (Table 1). On the contrary, the liver contained very low concentrations of imidacloprid. The pooled liver concentrations of the three Cape spurfowl, analysed in duplicate, were 16 ng/g and 29 ng/g wet weight.

**TABLE 1:** Imidacloprid concentrations (μg/g wet weight) in crop contents and liver samples collected from poisoned birds.

Sample	Current study^a^	Berny et al. (1999)^b^	Lopez-Antia et al (2015)^c^	Millot et al (2017)^b^
Crop content	7.160	11.4 (partridges) 16.3 (pigeons)	55.300 (high dose) 4.100 (low dose)	15.0 (partridges) 34.7 (pigeons)
Liver	0.016^d^ 0.029^d^	1.2 (partridges) 1.7 (pigeons)	0.083 (high dose) 0.056 (low dose)	3.0 (partridges) 1.4 (pigeons)

*Source*: Compiled by authors from the sources listed in the table.

Limit of quantitation (LOQ) = 10 ng/g.

a , Pooled samples of three Cape spurwing.

b , median imidacloprid concentration.

c , mean imidacloprid concentrations measured in red-legged partridges.

d , duplicate samples.

## Discussion

The neurological signs observed during this outbreak were similar to those reported by Millot et al. (2017). They described a sudden fall during flight, ataxia, paresis, paralysis and behavioural changes. In the current case, haemorrhage was present in two of the three birds within the coelomic cavity and is probably of traumatic origin considering the history that these birds fell from the sky. Congestion and haemorrhages in several organs were also reported by Millot et al. (2017). During this outbreak, the mortalities only occurred over a short period, which is in agreement with the transient effect previously observed (Millot et al. 2017).

Berny et al. (1999) and Millot et al. (2017) employed a high-performance thin layer chromatography (HPTLC) technique, and Lopez-Antia et al. (2015) measured imidacloprid concentrations by liquid chromatography–mass spectrometry (LC-MS). Detection with LC-HRMS is very sensitive, as indicated by the low concentrations that were measured (limit of quantitation [LOQ] = 10 ng/g) in this study. Because of the acuteness of mortality, Millot et al. (2017) surmised that imidacloprid might only be detectable in the crop or gizzard and not in the liver. Therefore, analysis using this highly sensitive LC-HRMS technique is advisable. Recovery of imidacloprid from the blank liver matrix at 50 ng/g was slightly higher at 130%.

Berny et al. (1999) and Millot et al. (2017) reported median imidacloprid concentrations of 11.4 *μ*g/g and *μ*g/g 16.3 and 15.0 *μ*g/g and 34.7 *μ*g/g in crop samples from grey partridge and pigeons, respectively. Lopez-Antia et al. (2015) determined mean concentrations of 4.1 *μ*g/g – 55.3 *μ*g/g in the crop contents of red-legged partridges (*Alectoris rufa*) that died during an experiment where they ingested imidacloprid-treated wheat seeds, at a lower (8.8 mg/kg/d) and higher (44 mg/kg/d) dosage rate.

The concentration of imidacloprid measured in this study was slightly lower than the mean liver imidacloprid concentrations of 56.0 ng/g – 82.6 ng/g wet weight reported by Lopez-Antia et al. (2015). Conversely, Berny et al. (1999) and Millot et al. (2017) reported much higher median liver concentrations of 1.2 *μ*g/g and 1.7 *μ*g/g and 1.4 *μ*g/g and 3 *μ*g/g wet weight, respectively, in grey partridge and pigeons found dead and suspected of being poisoned in France. The lower imidacloprid concentrations detected in this study could be ascribed to the longer interval from first sightings of neurobehavioural symptoms to when birds were eventually collected for necropsy examination or could imply that Cape spurfowl is more susceptible. Susceptibility to imidacloprid intoxication varies amongst different seed-eating bird species (Berny et al. 1999; Millot et al. 2017). This can be ascribed to the inherent seed-handling behaviour of granivorous birds, where some species swallow the seed whole and others discard the seed hulls (Avery, Fischer & Primus 1997). Two opinions expressed on factors that reduce the risk of imidacloprid ingestion by granivorous birds are the covering and burying of imidacloprid-treated seeds with soil after sowing or planting and natural aversion (Avery et al. 1994; Millot et al. 2017). However, these are not completely preventative, and treated seeds still pose a significant risk to granivorous birds (Millot et al. 2017) and in particular to Cape spurfowl as they scratch out planted seeds, which may increase their exposure. Spilled seeds or the failure to cover the seeds with soil could have contributed to this incident. Farmers should comply with the instructions and heed the warnings provided on labels and package inserts of commercially available products.

High-resolution mass spectrometry (HRMS) data can retrospectively be investigated for possible metabolites or degradation products based on accurate mass (5 ppm), matching isotope patterns with theoretical values or matching spectra with MS^2^ libraries. This can give some insight into metabolites where standard reference materials are not available, as indicated in Table 2. Some of the primary metabolites recognised in mammals are 4- and 5-hydroxyimidacloprid, 6-chloronicotinic acid, olefin, guanidine and urea derivatives (Wang et al. 2018). The Food and Agricultural Organization of the United Nations (FAO) (undated) lists 40 possible metabolites of imidacloprid. These compounds were included in the processing method and the results are listed in Table 2. The peak areas of the metabolites observed in the crop and liver samples are also provided in Table 2. Although peak area is not directly linked to concentration, it still provides a means to compare the metabolism of imidacloprid in the crop and liver. The crop sample contained mainly imidacloprid, which was ten times higher than any of the other peaks. The main metabolites detected in the crop sample were imidacloprid–nitrosimine, hydroxyimidacloprid, imidacloprid–urea and imidacloprid–denitro. The metabolites in the crop sample are the same as those found in the degradation pathway in soil (FAO, undated) and is possibly because of soil degradation rather than metabolism in the crop. Compared to the metabolism of imidacloprid in laying hens (FAO), hydroxylated, denitro and urea metabolites were also present in the Cape spurfowl samples. 6-Chloronicotinic acid, a major metabolite in mammals (Tomlin 2009), could not be detected in the liver, which is in agreement with observations reported by Berny et al. (1999), who could also only detect trace amounts. The imidacloprid–denitro–olefin metabolite (not reported in the laying hens) appears to be a major metabolite in the liver compared to the soil sample.

**TABLE 2:** Imidacloprid and its metabolites detected by liquid chromatography–high-resolution mass spectrometry in the crop contents and liver samples collected from Cape spurfowl that died in the Western Cape.

Metabolite nr^a^	Compound	Formula	Retention time (min)	Accurate mass [M+H^+^]	Crop peak area (cps)	Liver peak area (cps)
Parent	Imidacloprid^b^	C_9_H_10_ClN_5_O_2_	8.53	256.0595788	5.70 × 10^9^	3.10 × 10^7^
M01/M02	Hydroxyimidacloprid^b^	C_9_H_10_ClN_5_O_3_	7.72	272.0544934	1.83 × 10^8^	7.10 × 10^6^
M03	Dihydroxyimidacloprid^c^	C_9_H_10_ClN_5_O_4_	7.43	288.0494080	3.53 × 10^6^	3.62 × 10^6^
M06	Imidacloprid-olefin^b^	C_9_H_8_ClN_5_O_2_	7.43	254.0439288	6.20 × 10^7^	1.11 × 10^7^
M07	Imidacloprid-nitrosimine^c^	C_9_H_10_ClN_5_O	7.24	240.0646642	1.90 × 10^8^	ND
M08	Imidacloprid-amino^c^	C_9_H_12_ClN_5_	5.78	226.0853997	ND	5.46 × 10^6^
M09	Imidacloprid-denitro^b^	C_9_H_11_ClN_4_	4.34	211.0745007	1.63 × 10^8^	4.22 × 10^7^
M12	Imidacloprid-urea^b^	C_9_H_10_ClN_3_O	7.68	212.0585162	1.76 × 10^8^	1.23 × 10^7^
M13	Urea compound^c^	C_7_H_8_ClN_3_O	5.93	186.0428662	2.43 × 10^6^	8.77 × 10^6^
M14	6-Chloronicotinic acid^c^	C_6_H_4_ClNO_2_	8.12	158.0003326	8.51 × 10^5^	ND
M23	Imidacloprid-denitro-olefin^b^	C_9_H_9_ClN_4_	4.13	209.0588434	4.72 × 10^6^	1.32 × 10^8^
M31/M32	Keto-imidacloprid^c^	C_9_H_8_ClN_5_O_3_	9.0	270.0388434	4.24 × 10^6^	ND
M33/M34	NTN33896-diketone^c^	C_9_H_8_ClN_3_O_2_	7.19	226.0377808	8.78 × 10^6^	4.54 × 10^6^
M40	Formyl-AMCP^c^	C_7_H_7_ClN_2_O	6.38	171.0319671	3.94 × 10^7^	6.62 × 10^5^

cps, counts per second; ND, not detected.

a , Metabolite numbers were taken from http://www.fao.org/fileadmin/templates/agphome/documents/Pests_Pesticides/JMPR/Evaluation02/IMIDA_EVjjb.pdf.

b , confirmed with MS^2^ library spectrum.

c , identified using accurate mass and isotope ratios.

Some of the neonicotinoid toxic effects can be attributed to the induction of oxidative stress and the generation of reactive oxygen species (ROS) and reactive nitrogen species (RNS) (Lopez-Antia et al. 2015; Wang et al. 2018). In addition, exposure to lower concentrations of imidacloprid in birds can lead to sub-lethal effects such as decreased reproduction and impairment of the pituitary–thyroid axis (Lopez-Antia et al. 2015; Pandey & Mohanty 2015). Furthermore, it has been recorded that in the Netherlands even insectivorous bird populations are decreasing in areas with higher neonicotinoid concentrations in the surface water, but this is ascribed to a decrease in insect populations (Hallmann et al. 2014).

On 01 December 2013, the European Union placed a prohibition (EU Regulation 485/2013) on the marketing of imidacloprid as a seed treatment, albeit because of the deleterious effect on pollinators such as bees (Cresswell 2011; Lopez-Antia et al. 2015). However, regulatory authorities of countries in sub-Saharan Africa where imidacloprid is registered as a seed treatment should take note of the moratorium issued by the European Union and re-assess the risk to pollinators and vertebrates in Africa.
